# Mechanisms underlying the cardiac antifibrotic effects of losartan metabolites

**DOI:** 10.1038/srep41865

**Published:** 2017-02-03

**Authors:** José Luis Miguel-Carrasco, Javier Beaumont, Gorka San José, María U. Moreno, Begoña López, Arantxa González, Guillermo Zalba, Javier Díez, Ana Fortuño, Susana Ravassa

**Affiliations:** 1 Program of Cardiovascular Diseases, Centre for Applied Medical Research, University of Navarra, Pamplona, Spain; 2 Department of Physiology, University of Sevilla, Sevilla, Spain; 3 IdiSNA, Navarra Institute for Health Research, Pamplona, Spain; 4 CIBERCV, Carlos III Institute of Health, Madrid Spain; 5 Department of Biochemistry and Genetics, University of Navarra, Pamplona, Spain; 6 Department of Cardiology and Cardiac Surgery, University of Navarra Clinic, Pamplona, Spain

## Abstract

Excessive myocardial collagen deposition and cross-linking (CCL), a process regulated by lysyl oxidase (LOX), determines left ventricular (LV) stiffness and dysfunction. The angiotensin II antagonist losartan, metabolized to the EXP3179 and EXP3174 metabolites, reduces myocardial fibrosis and LV stiffness in hypertensive patients. Our aim was to investigate the differential influence of losartan metabolites on myocardial LOX and CCL in an experimental model of hypertension with myocardial fibrosis, and whether EXP3179 and EXP3174 modify LOX expression and activity in fibroblasts. In rats treated with N^G^-nitro-L-arginine methyl ester (L-NAME), administration of EXP3179 fully prevented LOX, CCL and connective tissue growth factor (CTGF) increase, as well as fibrosis, without normalization of blood pressure (BP). In contrast, administration of EXP3174 normalized BP and attenuated fibrosis but did not modify LOX, CCL and CTGF. In TGF-β_1_-stimulated fibroblasts, EXP3179 inhibited CTGF and LOX expression and activity with lower IC50 values than EXP3174. Our results indicate that, despite a lower antihypertensive effect, EXP3179 shows higher anti-fibrotic efficacy than EXP3174, likely through its ability to prevent the excess of LOX and CCL. It is suggested that the anti-fibrotic effect of EXP3179 may be partially mediated by the blockade of CTGF-induced LOX in fibroblasts.

Myocardial fibrosis is involved in the development of left ventricular (LV) dysfunction and clinically overt heart failure (HF) in hypertensive patients^1^,^2^,^3^. Diverse experimental studies indicate that collagen-dependent LV chamber stiffness is influenced not only by the amount of collagen fibers but also by the degree of collagen cross-linking (CCL) within the fibers, a process whereby collagen fibrils are covalently linked to one another by the enzyme lysyl oxidase (LOX), providing stiffness and resistance to degradation of the resulting fibers^4^,^5^,^6^,^7^. Of notice, recent clinical studies point to an excess of CCL as a major determinant of LV dysfunction and clinical outcomes in patients with HF of hypertensive etiology^1^,^8^,^9^,^10^. In this context, the reduction of myocardial LOX expression and CCL has been demonstrated to be associated with diminution of LV chamber stiffness in hypertensive patients with HF^2^.

The angiotensin II type 1 receptor antagonist losartan is a prodrug metabolized by the cytochrome-P450 pathway in the liver producing two metabolites: EXP3174 and EXP3179. EXP3174 is the final metabolite and the pharmacological blocker of the AT_1_ receptor by which losartan exerts its antihypertensive actions^11^. EXP3179 is an intermediate metabolite that has no AT_1_ receptor blocking properties and mediates a variety of AT_1_ receptor-independent, non-hemodynamic actions^12^,^13^,^14^,^15^. It is known that losartan reduces LV stiffness in hypertensive patients, an effect associated with the reduction of the content of myocardial collagen fibers^16^. However, there is no information on the effects of this drug on myocardial LOX and CCL in hypertension. Therefore, we have investigated the effects of EXP3179 and EXP3174 on fibrosis, and LOX and CCL in the myocardium of rats with arterial hypertension induced by the NG-nitro-L-arginine methyl ester (L-NAME), an experimental model characterized by myocardial transforming growth factor-β_1_ (TGF-β_1_) overexpression and fibrosis^17^,^18^,^19^. In addition, we aimed to explore whether losartan metabolites influence LOX expression and activity in fibroblasts stimulated with TGF-β_1_.

## Results

### *In Vivo* Findings

#### Effects of Losartan Metabolites on Blood Pressure (BP)

**Figure 1:**
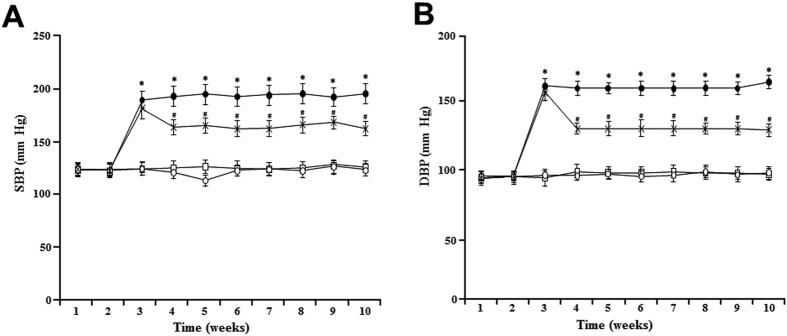
Time-course changes in systolic blood pressure (SBP) (panel A) and diastolic blood pressure (DBP) (panel B) in normotensive controls rats (□), L-NAME-treated rats (●), L-NAME + EXP3179-treated rats (x) and L-NAME + EXP3174-treated rats (○). Data points represent mean ± SEM (n = 5). *P < 0.01 vs Control and L-NAME + EXP3174, ^#^P < 0.05 vs L-NAME and Control.

From the third week until the end of treatment, systolic and diastolic blood pressure (SBP and DBP) were elevated in L-NAME rats compared to control normotensive rats (Fig. 1). L-NAME + EXP3179 rats exhibited reduced SBP and DBP as compared with L-NAME rats, although BP values remained significantly increased as compared to control normotensive rats (Fig. 1A,B). On the other hand, L-NAME + EXP3174 rats showed SBP and DBP values similar to control normotensive rats throughout the entire 10-week treatment period (Fig. 1A,B). Treatment with either EXP3179 or EXP3174 in the absence of L-NAME did not influence either SBP or DBP (data not shown).

#### Effects of losartan metabolites on LV morphology and function

As shown in Table 1, compared to control normotensive rats, L-NAME rats exhibited LV hypertrophy, as indicated by the increased relative wall thickness (RWT) and LV mass index (LVMI). The LVMI was normalized in L-NAME + EXP3179 rats but remained increased in L-NAME + EXP3174 rats (Table 1).

In addition, Table 1 shows that L-NAME rats exhibited reduced LV systolic (e.g., reduced ejection fraction and fractional shortening) and diastolic (e.g., reduced E/A ratio) function compared to control normotensive rats. Co-treatment of L-NAME rats with either EXP3179 or EXP3174 prevented these alterations (Table 1). None of the parameters assessing LV morphology and function were modified in rats treated with either metabolite alone (data not shown).

#### Effects of Losartan Metabolites on Myocardial TGF-β_1_ and CTGF Expression

**Figure 2:**
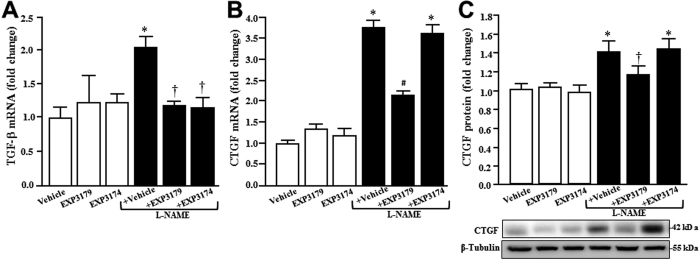
Histograms represent the expression of cardiac TGF-β_1_ (panel A) and CTGF (panel B) mRNA and CTGF protein (panel C) in the myocardium of Vehicle-, EXP3179-, EXP3174-, L-NAME + Vehicle-, L-NAME + EXP3179- and L-NAME + EXP3174-treated rats. Representative Western blot autoradiograms for CTGF are presented at the bottom of panel C. Bars represent mean + SEM (n = 10). *P < 0.05 vs Vehicle, EXP3179 and EXP3174, ^†^P < 0.05 vs L-NAME + Vehicle, ^#^P < 0.05 vs Vehicle and L-NAME + Vehicle.

Compared to control normotensive rats, L-NAME rats exhibited increased expression of myocardial TGF-β_1_ mRNA, which was fully prevented in L-NAME + EXP3179 and in L-NAME + EXP3174 rats (Fig. 2A). In addition, L-NAME rats showed increased expression of CTGF mRNA (Fig. 2B) and protein (Fig. 2C) compared to control normotensive rats. Co-treatment of L-NAME rats with EXP3179, but not with EXP3174, significantly decreased CTGF mRNA and protein expression induced by L-NAME (Fig. 2B,C, respectively). Treatment with either EXP3179 or EXP3174 in the absence of L-NAME did not exert any effect on these parameters (Fig. 2).

#### Effects of Losartan Metabolites on Myocardial Collagen Synthesis and Accumulation

**Figure 3:**
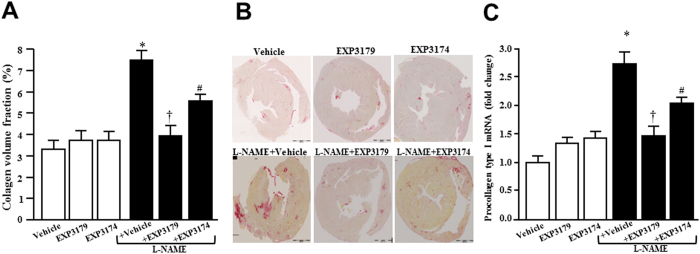
Histograms represent the percentage of collagen volume fraction (panel A) in the myocardium of Vehicle-, EXP3179-, EXP3174-, L-NAME + Vehicle-, L-NAME + EXP3179- and L-NAME + EXP3174-treated rats. Representative images of myocardial tissue from one rat of each group are shown in panel B. Sections were stained with picrosirius red and collagen fibers were identified in red. Expression of procollagen type I mRNA is shown in panel C. Bars represent mean + SEM (n = 10). *P < 0.05 vs Vehicle, EXP3179 and EXP3174, ^†^P < 0.05 vs L-NAME + Vehicle, ^#^P < 0.05 vs Vehicle and L-NAME + Vehicle.

Collagen volume fraction (CVF) was increased in L-NAME rats compared to control normotensive rats, being normal in L-NAME + EXP3179 rats but still abnormally increased in L-NAME + EXP3174 rats (Fig. 3A,B). Compared to control normotensive rats, L-NAME rats exhibited increased procollagen type I expression in cardiac tissue, which was fully prevented in L-NAME + EXP3179 rats and only partially prevented in L-NAME + EXP3174 rats (Fig. 3C). None of these parameters were modified in rats treated with either EXP3179 or EXP3174 in the absence of L-NAME (Fig. 3).

**Figure 4:**
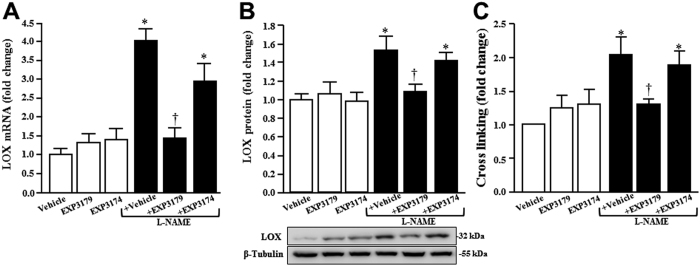
Histograms represent the fold change in the expression of LOX mRNA (panel A), LOX protein (panel B) and collagen cross-linking (panel C) in the myocardium of Vehicle-, EXP3179-, EXP3174-, L-NAME + Vehicle-, L-NAME + EXP3179- and L-NAME + EXP3174-treated rats. Representative Western blot autoradiograms for LOX protein expression are presented at the bottom of panel B. Bars represent mean + SEM (n = 10). *P < 0.05 vs Vehicle, EXP3179 and EXP3174, ^†^P < 0.05 vs L-NAME + Vehicle.

In addition, the myocardium of L-NAME rats exhibited increased LOX mRNA (Fig. 4A) and protein (Fig. 4B) expression, as well as an excess of CCL (Fig. 4C). These alterations were fully prevented in L-NAME + EXP3179 rats but not in L-NAME + EXP3174 rats. Treatment with either EXP3179 or EXP3174 in the absence of L-NAME did not exert any effect on these parameters (Fig. 4).

### *In Vitro* Findings

#### Effects of Losartan Metabolites on Procollagen Type I Expression, and LOX Expression and Activity in TGF-β_1_-stimulated adult human dermal fibroblasts (HDFa)

As it has been previously published for cardiac fibroblast^20^, incubation of HDFa with TGF-β_1_ for 24 h induced a dose-dependent increase in both procollagen type I mRNA (see Supplementary Fig. S1A) and LOX (see Supplementary Fig. S1B) expression. TGF-β_1_ 10^−4^ μg/mL was selected as the lowest concentration with submaximal effects on these parameters (see Supplementary Fig. S1).

**Figure 5:**
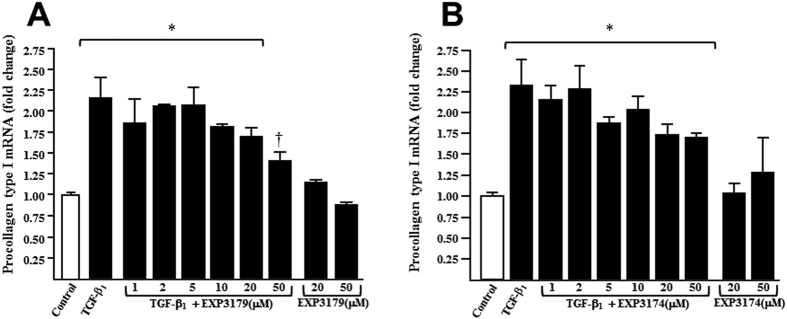
Histograms represent the fold change in procollagen type I mRNA expression in HDFa fibroblasts stimulated without and with 10^−4^ μg/mL TGF-β_1_ for 24 hours, in the absence or the presence of 1, 2, 5, 10, 20 and 50 μM of EXP3179 (panel A) and EXP3174 (panel B), or incubated with the two compounds alone at 20 and 50 μM, as compared with controls cells. Bars represent mean + SEM (n = 5 to 8 experiments). *P < 0.05 vs Control, ^†^P < 0.05 vs TGF-β_1_.

HDFa fibroblasts were incubated with 10^−4^ μg/mL TGF-β_1_ in the presence of EXP3179 and EXP3174 at increasing concentrations. TGF-β_1_-induced procollagen type I expression was significantly inhibited by EXP3179 (Fig. 5A) and it tended to be reduced by EXP3174 (Fig. 5B).

**Figure 6:**
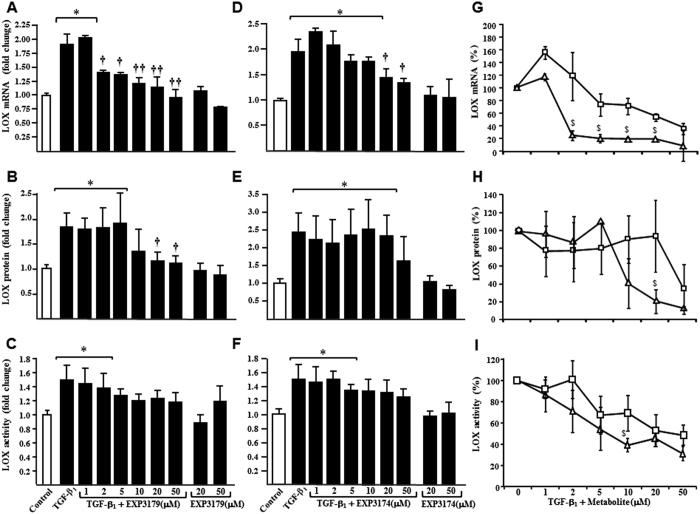
Histograms depict the fold change in LOX mRNA, protein and activity in HDFa fibroblasts stimulated without and with 10^−4^ μg/mL TGF-β_1_ for 24 hours, in the absence or the presence of 1, 2, 5, 10, 20 and 50 μM of EXP3179 (panels A–C) and EXP3174 (panels D–F), or incubated with the two compounds alone at 20 and 50 μM, as compared to control cells. Line graphs show percentage of inhibition in LOX mRNA (panel G), protein (panel H), and activity (panel I), in HDFa stimulated with 10^−4^ μg/mL TGF-β_1_ in combination with EXP3174 (□) or EXP3179 (∆) at the above mentioned concentrations, compared to cells incubated with TGF-β_1_ alone. Bars represent mean +SEM (n = 5 to 8 experiments) and curves show means ± SEM (n = 5 to 8). *P < 0.05 vs Control, ^†^P < 0.05 vs TGF-β_1_, ^††^P < 0.01 vs TGF-β_1_ and ^$^P < 0.05 vs EXP3174.

In addition, EXP3179 inhibited TGF-β_1_ effects on LOX mRNA (Fig. 6A) and protein (Fig. 6B) expression, with a clear tendency to inhibit extracellular LOX activity (Fig. 6C). Moreover, EXP3174 inhibited TGF-β_1_-induced LOX mRNA expression (Fig. 6D) and tended to reduce TGF-β_1_-induced LOX protein (Fig. 6E) and activity (Fig. 6F) only at high doses.

For all LOX parameters, EXP3179 was identified as a more potent inhibitor as compared to EXP3174 (Fig. 6G–I), with lower IC50 values than EXP3174 (LOX mRNA: 1.6 ± 0.1 vs 29.4 ± 1.5 μM, P < 0.001; LOX protein: 8.9 ± 1.0 vs 38.5 ± 5.0 μM, P < 0.05; LOX activity: 7.4 ± 0.3 vs 37.3 ± 2.9 μM, P < 0.05).

#### Involvement of Intracellular Pathways on EXP3179-mediated Inhibition of LOX Expression

We investigated whether classical intracellular pathways involved in the actions described for EXP3179 in other experimental settings^12^,^13^,^14^,^15^ were also involved in its inhibitory effects on TGF-β_1_-induced LOX upregulation in HDFa fibroblasts. However, none of the compounds tested (i.e. the inhibitors of PPAR-γ [G3335] and PI3K [LY294002], and the activators of PKC [PMA] and COX [LPS]) were able to counteract the actions of EXP3179 on LOX mRNA expression (see Supplementary Fig. S2).

In addition, we analysed in HDFa fibroblasts the involvement of diverse fibrosis-related genes in the EXP3179-induced LOX inhibition, by using the RT2 Profiler^TM^ PCR Array Human Fibrosis. As shown in table S1, 84 genes were examined, confirming the TGF-β_1_-induced increment in LOX expression and its inhibition in the presence of EXP3179, both exceeding a 1.5 fold change. Therefore, this threshold was chosen to select those genes overexpressed by TGF-β1 that were inhibited by EXP3179. According to the array results and following the proposed criteria, CTGF and thrombospondin-1 (THBS1) were selected as genes that were potentially involved in the anti-fibrotic actions of EXP3179 (Supplemental Table S1). The inhibitory actions of EXP3179 on these genes were confirmed by real-time RT-PCR (data not shown). Therefore, CTGF and THBS1 genes were selected for further analyses.

By silencing CTGF expression with siRNA in HDFa, TGF-β_1_-induced LOX expression was inhibited. On the contrary, siRNA-mediated THBS1 inhibition did not exert any influence on this pathway (Supplementary Fig. S3A). In addition, we observed that, as in the case of LOX, EXP3179 inhibited CTGF mRNA expression in a dose-dependent manner (see Supplementary Fig. S3B).

## Discussion

The main findings of this study are the following: 1) Chronic inhibition of NO synthesis with L-NAME in Wistar rats resulted in a hypertensive model of myocardial fibrosis with increased expression of CTGF and LOX, as well as enhanced CCL; 2) Administration of EXP3179 in L-NAME rats exerted a partial anti-hypertensive effect, whereas EXP3174 fully prevented the increase in BP; 3) Whereas the administration of EXP3179 in L-NAME rats fully prevented myocardial CTGF and LOX overexpression, and excessive CCL and fibrosis, EXP3174 administration was not able to prevent LOX overexpression and CCL, and only partially reduced myocardial fibrosis; and 4) EXP3179 inhibited the TGF-β_1_-induced upregulation of LOX expression and activity in fibroblasts, probably through CTGF regulation, in a more effective manner than EXP3174.

The evidence supports that L-NAME-induced hypertension is a well-established experimental model, representative of the left ventricular remodeling, and specifically of the myocardial fibrosis, occurring in hypertensive heart disease^17^,^18^,^19^,^21^,^22^,^23^,^24^,^25^. In addition, Tsukamoto *et al*. further characterized this model demonstrating that the lack of NO is associated with impairment of LV systolic and diastolic function^22^. In this regard, we confirm these findings and expand the characterization of myocardial fibrosis in this model with novel data demonstrating that reduced NO bioavailability is associated with increased myocardial LOX expression and enhanced cross-linking of collagen fibrils.

Although several clinical^16^,^26^,^27^ and experimental studies^28^,^29^,^30^ have demonstrated that losartan prevents and/or regresses the myocardial fibrosis associated with arterial hypertension, the role of losartan metabolites in this anti-fibrotic effect has not been characterized. In this regard, we report here that administration of EXP3179 completely prevented the excess of CTGF, LOX and CCL, and fully abrogated myocardial fibrosis, yet without normalization of BP. In contrast, we found that although administration of EXP3174 normalized BP, it failed to prevent myocardial CTGF and LOX overexpression, as well as the excess of CCL, and only partially reduced myocardial fibrosis. Of notice, both treatments prevented LV systolic and diastolic dysfunction. Therefore, we may speculate that EXP3179 may improve LV function by inhibiting CTGF-dependent pro-fibrotic mechanisms in cardiac fibroblasts, with a mild anti-hypertensive effect, whereas EXP3174 may improve LV function through normalization of BP with a concomitant, mild inhibitory effect on myocardial fibrosis.

In order to further evaluate the potential molecular mechanisms involved in the differential anti-fibrotic effects of losartan metabolites, *in vitro* experiments were performed. In accordance with previous studies^20^,^31^, we observed an upregulation of procollagen type I and LOX expression and activity in fibroblasts stimulated with TGF-β_1_. In addition, we observed that CTGF mediates TGF-β_1_-induced LOX expression, confirming previous studies in which such an association was suggested^32^. Moreover, we report that EXP3179 showed a higher efficacy than EXP3174 in reducing LOX expression in HDFa, with significant effects at doses similar to those present in blood from patients chronically treated with losartan (~2μM)^33^. In this regard, examination of a large number of genes in the fibrosis pathway and the *in vitro* experiments with specific siRNAs revealed that EXP3179 effects on LOX are likely mediated through downregulation of CTGF. Our results provide mechanistic support to previous findings demonstrating that losartan is able to interfere with the profibrotic activity of TGF-β_1_, including downregulation of CTGF^34^,^35^,^36^. In this regard, since CTGF may be a critical mediator of the EXP3179 actions on LOX expression, evaluation of different intracellular pathways participating in the regulation of CTGF (e.g., the c-Jun N-terminal kinase [JNK] pathway)^37^,^38^ could be considered for future studies. This is of particular interest taking into account that we observed that rats co-treated with L-NAME and EXP3174 exhibited CTGF overexpression despite TGF-β_1_ being normalized, with a mild anti-fibrotic effect. In this regard, the TGF-β_1_-independent upregulation of CTGF and collagen synthesis induced by angiotensin II has been reported in different *in vivo* models of renal damage^39^, and in atrial fibrillation^40^. In addition, other mechanisms besides TGF-β_1_, and related to the renin-angiotensin system, hemodynamic stress^41^ or inflammation/oxidative stress^17^, have been reported as inductors of myocardial fibrosis in L-NAME-treated rats. On the other hand, the mild antifibrotic effect shown by EXP3174 may be due to abrogation of chronic hemodynamic stress, independently of CTGF. This notion is supported by previous studies demonstrating that treatment with losartan or hydrochlorothiazide showed similar anti-fibrotic and anti-hypertensive effects in the myocardium of SHR rats receiving high salt diet, being losartan more effective in decreasing CTGF expression^42^.

Some limitations of the present study must be acknowledged. First, a losartan group was not included in the study. Nevertheless, the aim of the study was to examine the effects of both metabolites on myocardial fibrosis present in L-NAME rats, in order to determine their individual anti-fibrotic actions on the hypertensive myocardium. Second, few studies have applied EXP3179 as an individual potential drug and therefore a dose-dependent pharmacological profile of EXP3179 has not been established yet. Third, further experiments are needed to analyse the myocardial distribution of extracellular matrix/profibrotic proteins in the L-NAME-induced hypertension model. Fourth, in the *in vitro* study, the potential contribution of the remaining differentially expressed genes in the array was not analysed in depth. In addition, further studies are required to confirm these findings in human cardiac fibroblasts. Finally, it is unknown whether the anti-fibrotic effects of EXP3179 were solely due to prevention of collagen synthesis and deposition or if the metabolite also influences the enzymes that control collagen degradation.

We conclude that despite a lower hypertensive efficacy, the losartan metabolite EXP3179 is more effective improving myocardial fibrosis than EXP3174 in L-NAME rats. In particular, we found that EXP3179 is more efficient than EXP3174 downregulating LOX expression, with these effects resulting in a normalization of CCL. Of interest, these molecular and anti-fibrotic effects of EXP3179 in L-NAME rats may be mediated by the inhibition of the TGF-β_1_-CTGF pathway that is activated in the myocardium of these rats.

## Methods

For detailed description, see Methods in the online Data Supplement.

### *In Vivo* Procedure

Forty five 10-week-old male Wistar rats were treated for 10 weeks, once-daily, with 30 mg/kg of L-NAME by oral administration, a dose sufficient to induce arterial hypertension^43^ and 45 untreated rats were considered as controls. Fifteen animals in each group were co-treated, once-daily, with either vehicle, EXP3179 or EXP3174 by oral administration. EXP3174 was administered at 5 mg/kg/day, a dose resulting in circulating metabolite levels similar to those found in patients chronically treated with losartan^33^,^44^. EXP3179 was administered at the same dose. Blood pressure (BP) was assessed in five animals from each group by radiotelemetry monitoring.

### Echocardiographic Studies

Echocardiography was performed using a Vevo 770 ultrasound system. The heart rate (HR) of the animals was recorded immediately before the echocardiographic study.

### Collagen Cross-linking Analysis

The evaluation of the degree of CCL was performed using Fast Green-Sirius Red and Sircol-based colorimetric assays in myocardial tissue.

### Assessment of Collagen Volume Fraction

CVF was determined in heart sections of rats as a percentage of total myocardial area occupied by collagen tissue.

### *In Vitro* Procedure

After carrying out dose-response curves to human recombinant TGF-β_1_ (RαD systems), adult human dermal fibroblasts (HDFa line; GIBCO) were incubated for 24 hours with or without 10^−4^ μg/mL, in the absence or presence of the metabolites EXP3174 and EXP3179 (provided by Merck & Co, Inc.) at a range of concentrations. In addition, HDFa cells were co-incubated with EXP3179 in the absence or presence of the following compounds: the inhibitors of PPAR-γ (G3335) and phosphatidylinositol 3-kinase (PI3K) pathway (LY294002) and the activators of protein kinase-C (PKC) (Phorbol 12-myristate 13-acetate [PMA]) and cyclo-oxygenase-2-pathway (lipopolysaccharide [LPS]).

### Human fibrosis array

Samples from cells incubated in control conditions or with TGF-β_1,_ in the absence or presence of EXP3179 (20μM) were examined by using the Human Fibrosis RT2 Profiler PCR Array (SABiosciences Corp.) consisting of a panel of 84 key genes involved in fibrosis.

### Quantitative Real-time PCR and Gene Silencing

Gene expression was analysed by quantitative real-time PCR by using specific TaqMan fluorescent probes. Expression of connective tissue growth factor (CTGF) and thrombospondin 1 (THBS1) was silenced by small interfering RNAs in HDFa.

### Assessment of Protein Expression and Evaluation of LOX Activity

The expression of several proteins was analysed by Western Blot. LOX activity was assessed by a commercially available fluorimetric assay.

### Statistical Analysis

Variables are expressed as means ± SEM. Differences among different conditions were tested by one-way ANOVA followed by the Fisher’s least significant difference method for post-hoc comparisons once normality was checked (Shapiro-Wilks test); otherwise, the nonparametric Kruskal-Wallis test followed by a Mann-Whitney U test (adjusting the α-level by Bonferroni inequality) was used. Statistical significance was defined as two-sided P < 0.05. The analyses were performed using the programs SPSS (15.0 version) and STATA (12.1 version).

### Ethical standards

All authors in this work gave their informed consent prior to their participation in the study. The manuscript does not contain clinical studies or patient data. The research conformed to the Guide for the Care and Use of Laboratory Animals published by the US National Institutes of Health (NIH Publication No 85–23, revised 1996), and was approved by the Ethical Committee for Animal Experimentation of the University of Navarra (036/08).

## Additional Information

**How to cite this article**: Miguel-Carrasco, J. L. *et al*. Mechanisms underlying the cardiac antifibrotic effects of losartan metabolites. *Sci. Rep.* **7**, 41865; doi: 10.1038/srep41865 (2017).

**Publisher's note:** Springer Nature remains neutral with regard to jurisdictional claims in published maps and institutional affiliations.

## Supplementary Material

Supplementary Information

## Figures and Tables

**Table 1:** Echocardiographic Parameters.

Variables	Vehicle	L-NAME		
		+Vehicle	+EXP3179	+EXP3174
LV morphology				
RWT	0.42 ± 0.01	0.53 ± 0.02**	0.49 ± 0.02*	0.52 ± 0.01**
LVMI, mg/g	1.32 ± 0.05	1.82 ± 0.06**	1.45 ± 0.02^††^	1.68 ± 0.02**^†^
LV systolic function				
EF, %	72.3 ± 0.83	63.1 ± 3.35*	75.6 ± 2.35^†^	73.5 ± 3.28^†^
FS, %	43.7 ± 0.45	35.1 ± 2.34*	45.7 ± 2.14^†^	44.1 ± 2.70^†^
LV diastolic function				
E, cm/s	81.1 ± 5.19	69.4 ± 3.52*	82.8 ± 2.03^†^	83.1 ± 4.25^†^
A, cm/s	58.2 ± 3.51	60.6 ± 1.13	62.2 ± 3.02	58.4 ± 1.53
E/A	1.40 ± 0.06	1.19 ± 0.03*	1.35 ± 0.07^†^	1.43 ± 0.08^†^

Data are expressed as mean ± SEM. LV means left ventricle; RWT, relative wall thickness; LVMI, left ventricular mass index; EF, ejection fraction; FS, fractional shortening; E, peak velocity of early transmitral inflow; A, peak velocity of late transmitral inflow. *P < 0.05 vs Vehicle; ^†^P < 0.05 vs L-NAME + Vehicle; **P < 0.01 vs Vehicle; ^††^P < 0.01 vs L-NAME + Vehicle.
