# Supplementary material for: Mechanisms underlying the cardiac antifibrotic effects of losartan metabolites
Source: Sci Rep. 2017 Feb 3;7:41865. doi: 10.1038/srep41865 (PMC5291109; doi:10.1038/srep41865)
Supplement: Supplementary Information [file srep41865-s1.pdf]

ONLINE DATA SUPPLEMENT

Mechanisms underlying the cardiac antifibrotic effects of losartan metabolites

José Luis Miguel-Carrasco[1,2]*, Javier Beaumont[1,3]* Gorka San José[1,3], María U Moreno[1,3], Begoña López[1,3], Arantxa González[1,3], Guillermo Zalba[1,3,4], Javier Díez[1,3,5], Ana Fortuño[1] and Susana Ravassa[1,3]

*Both authors contributed equally to this work

[1]Program of Cardiovascular Diseases, Centre for Applied Medical Research, University of Navarra, Pamplona, Spain; [2]Department of Physiology, University of Sevilla, Sevilla, Spain; [3]IdiSNA, Navarra Institute for Health Research, Pamplona, Spain; [4]Department of Biochemistry and Genetics, University of Navarra, Pamplona, Spain; [5]Department of Cardiology and Cardiac Surgery, University of Navarra Clinic, Pamplona, Spain.

Corresponding author:

Ana Fortuño, Ph.D., Program of Cardiovascular Diseases, CIMA, Avenida Pío XII 55, 31008 Pamplona, Spain. E-mail afortuno@unav.es; Phone: (34) 948194700; Fax: (34)948194716.

and

Susana Ravassa, Ph.D., Program of Cardiovascular Diseases, CIMA, Avenida Pío XII 55, 31008 Pamplona, Spain. E-mail sravassa@unav.es; Phone: (34) 948194700; Fax: (34)948194716.

Methods

In Vivo Study

Experimental Design

The research conformed to the Guide for the Care and Use of Laboratory Animals published by the US National Institutes of Health (NIH Publication No 85–23, revised 1996)[1], and was approved by the Ethical Committee for Animal Experimentation of the University of Navarra (036/08). Rats were provided by Harlan UK Limited (Bicester, UK). Ten weeks old male Wistar rats were assigned into six groups of 15 animals each: (1) control group receiving the vehicle solution (vehicle), (2) control group treated with EXP3179 (EXP3179), (3) control group treated with EXP3174 (EXP3174), (4) hypertensive group treated with L-NAME (L-NAME), (5) L-NAME treated rats subjected to simultaneous treatment with EXP3179 (L-NAME+EXP3179), and (6) L-NAME treated rats subjected to simultaneous treatment with EXP3174 (L-NAME+EXP3174). The treatment with L-NAME consisted on a dose of 30 mg of L-NAME/kg body weight/day dissolved in the drinking water, which is reported to induce hypertension[2]. The treatment with losartan metabolites EXP3179 or EXP3174 consisted in a daily oral administration dose of 5 mg/kg body weight/day using gavage for 10 weeks. According to previous studies, this dose of EXP3174 results in circulating metabolite levels similar to those found in patients chronically treated with losartan[3,4]. Treated rats were housed in cages with free access to standard rat chow and tap water in a quiet room with constant temperature (20–22ºC) and humidity (50–60%). Before they were sacrificed by decapitation, the rats were weighed and anaesthetized using isoflurane (IsofloH, ABBOTT S.A.) at a concentration of 4% (induction) in oxygen 100%.

Five animals from each group were used for radiotelemetry monitoring of blood pressure. Rats were administered buprenorphine (0.1 mg/kg SC) before surgery and anesthetized with isoflurane at a concentration of 4% (induction) and 1.5–2% (maintenance) in oxygen 100%. A flexible catheter was secured into the abdominal aorta and a telemetry transmitter was sutured to the abdominal wall. On recovery, rats were housed in individual cages, and each cage was placed over a receiver panel with output to a computer. After a 1-week recovery period, heart rate (HR), SBP and DBP were recorded for 10 seconds every 5 minutes once a week. Twelve-hour day and night mean values were computed (Dataquest IV, Data Sciences International).

Echocardiographic Studies

Echocardiography was performed using a Vevo 770 ultrasound system (Visualsonics, Toronto, Canada) equipped with a real time micro-visualization scan head probe (RMV-710 B) working at a frame rate ranging between 110 and 120 frames per sec (fps). The nosepiece-transducer used has a central frequency of 25 MHz, a focal length of 15 mm and 70 mm of nominal spatial resolution. Rats were anesthetized with isoflurane, at a concentration of 4% (induction) and 2% (maintenance) in 100% oxygen. Each animal was placed on a heating table in a supine position with the extremities tied to the table through four electrocardiography leads. The chest was shaved using a chemical hair remover (Veet, Reckitt Benckise,

Granollers, Spain). Warmed ultrasound gel (Quick Eco-Gel, Lessa, Barcelona, Spain) was applied to the thorax surface to optimize the visibility of the cardiac chambers. The heart rate (HR) of the animals was recorded immediately before the echocardiographic study.

Preparation of Tissue Samples

After sacrifice, hearts were carefully excised, dissected out and cut into pieces. One piece was frozen at -80ºC for mRNA and protein analysis. Another piece for immunohistochemistry and histomorphological studies was immediately fixed in 4% buffered formalin, embedded in paraffin, and serially sectioned in 4 µm thick sections with a rotation microtome.

Histomorphological Analysis

Histological evaluation was performed by two independent observers in a blinded fashion. Heart sections were stained with collagen-specific picrosirius red (Sirius red F3BA in aqueous picric acid) according to Dolber and Spach[5]. Collagen volume fraction (CVF) was determined as the percentage of total area occupied by collagen by quantitative morphometry with an automated image analysis system (AnalySYS 3.1, Soft Imaging System GmbH, Hammer, Germany). The evaluation of cardiac fibrosis was performed in a whole myocardial slide from each rat (n=10 animals per group).

Cross-linking Analysis

According to our previous experience[6], to evaluate the degree of cross-linking of collagen molecules to form collagen fibers, the amounts of cross-linked (insoluble) and non-cross-linked (soluble) collagen were determined using two colorimetric assays: Fast Green-Sirius Red to obtain total collagen and Sircol-based assay to obtain soluble collagen. Insoluble collagen was calculated as the difference between total collagen and soluble collagen. The degree of cross-linking was calculated as the ratio between the insoluble and the soluble forms of collagen, and expressed as percentage with respect to controls.

Real-time RT-PCR

Total RNA was extracted from frozen heart sections automatically by the ABI PRISM® 6100 Nucleic PrepStation (Applied Biosystems) method. 500 ng of total RNA were reversed transcribed to cDNA using the RNA SuperScript® VILOTM cDNA Synthesis Kit (Invitrogen). Real time PCR analyses were performed as previously described with probes for procollagen type I (Rn 01463848), TGF-β_1 (Rn 99999016), connective tissue growth factor (CTGF, Rn 00573960), lysyl oxidase (LOX, Rn 01491829) and 18S ribosomal RNA (Hs 03003631). Data were normalized to 18S ribosomal RNA expression. Relative expression of mRNA was determined by the comparative $2^{-\Delta\Delta CT}$ method[7].

Western blot

To analyze the expression of CTGF, PCP, pro-collagen C-proteinase enhancer (PCPE) and LOX proteins, heart homogenates were separated by electrophoresis in polyacrylamide gels and transferred onto nitrocellulose membranes. Specific rabbit antibodies were incubated overnight at 4ºC (CTGF, Torrey

Pines, 1:10000; PCP, Abcam, Cambridge, UK 1:1000; PCPE, Santa Cruz, 1:500; LOX zymogen and active form, Abcam, Cambridge, UK, 1:100). Primary antibodies were detected by incubation with appropriate peroxidase-conjugated secondary antibodies (horseradish peroxidase-conjugated secondary antibody (GE Healthcare UK limited)) for 1 hour at room temperature (1:20000 anti-rabbit for CTGF, 1:5000 anti-rabbit for PCP, 1:2500 anti-goat for PCPE and 1:5000 anti-rabbit for LOX). Protein expression was visualized with the ECL-Advanced chemiluminiscence system (Amersham Biosciences). Membranes were analyzed using the Chemidoc Detection System and analyzed by Quantity One software (Bio-Rad) obtaining arbitrary densitometric units (ADUs). The blots were reprobed with a monoclonal β-tubulin antibody (Sigma) to normalize for loading.

In Vitro Study

HDFa Cell Culture

Adult human dermal fibroblasts (HDFa) (Thermo Fisher Scientific) were grown in DMEM medium (Thermo Fisher Scientific) supplemented with 10 % fetal bovine serum and fibroblast growth factor. Cells were left to expand until they reached 60% confluency and then starved in free-serum medium for 24h. After preliminary dose-response curves in response to different concentrations of TGF-β_1 (RαD systems) (Figure S1), cells were then incubated with or without 10^{-4} μg/ml human recombinant TGF-β_1 (RαD systems) in the absence or presence of the metabolites EXP3174 and EXP3179 (provided by Merck & Co, Inc) at 1, 2, 5, 10, 20 and 50 μM for 24 hours. In addition, HDFa cells were co-incubated with TGF-β_1 and 20μM EXP3179 in the absence or presence of the following compounds: the inhibitor of PPAR-γ, G3335 (at 32 and 100 μM; Santa Cruz Biotechnology), the inhibitor of the phosphatidylinositol 3-kinase (PI3K) pathway, LY294002 (at 1.4 and 10 μM; Life Technologies), the activator of the protein kinase-C (PKC), Phorbol 12-myristate 13-acetate (PMA) (at 0.4 and 10 μM; Sigma) and the activator of the cyclo-oxygenase-2-pathway, lipopolysaccharide (LPS) (at 1 and 50 μg/mL; Sigma).

Human fibrosis array

We analyzed the profile of 84 genes involved in fibrosis with the RT^2 Profiler PCR Array Human Fibrosis (SABiosciences Corp.) in samples from control, TGF-β_1 and TGF-β_1+EXP3179-stimulated human adult fibroblasts. 400 ng of total RNA isolated from fibroblasts were reverse transcripted to cDNA using the RT^2 First Strand Kit (Sabioscience), and the cDNA was mixed with RT^2 SYBR Green Mastermix and dispensed into the RT^2 Profiler PCR Array and amplified, according to manufacturer's recommendations.

Quantitative Real-time PCR

mRNA levels were analysed in cell lysates by real-time quantitative PCR. Reverse transcription was performed with 800 ng of total RNA by using Superscript III reverse transcriptase (Invitrogen). Real-time PCR was performed with a 7900 HT Fast Real-time PCR system according to the manufacturer's recommendations (Life Technologies) by using specific TaqMan MGB fluorescent probes for human LOX (Hs 00942480_m1, Life Technologies), procollagen type I (Hs 00164004_m1), CTGF (Hs

00170014_m1, Life Technologies), THBS1 (Hs00962908_m1) and a specific TaqMan MGB fluorescent probe for human constitutive 18S ribosomal RNA (Hs 03003631) as endogenous control (Life Technologies). Data were analysed as arbitrary units (A.U.) relative to 18S ribosomal RNA.

Analysis of LOX Protein Expression and Activity

The expression and activity of LOX protein was analysed in the supernatant of HDFa cells. Supernatant was concentrated with Amicon Ultra-4 centrifugal filter units (Merck Millipore), and 15-20 μg of total protein obtained from cell supernatants were separated on 12% polyacrylamide gel and transferred onto nitrocellulose membranes. A LOX-specific polyclonal antibody (Abcam) at a dilution of 1:2500 was used to analyse the 36kDa form. Protein expression was visualized with the Lumigen ECL Ultra solution (Lumigen Inc). LOX activity was measured with a commercially available fluorimetric assay (AAT Bioquest) following the manufacture's indications as previously described[8]. All determinations performed in the supernatant were corrected by total protein amount.

CTGF RNA Silencing

CTGF and THBS1 silencing was performed using lipofectamine RNAiMax (Invitrogen) and 10 pmol/mL of siRNA (Silencer Select siRNA, Ambion) in HDFa fibroblasts following the manufacturer's instructions, obtaining 69% and 72% inhibition after 24 h, respectively.

References

1. Guide for the Care and Use of Laboratory Animals. National Research Council (US) Institute for Laboratory Animal Research. Washington, DC, USA. (1996).
2. Morán A., de Urbina A.V., Martín M.L., Rodríguez-Barbero A., Román L.S. Characterization of the contractile 5-hydroxytryptamine receptor in the autoperfused kidney of L-NAME hypertensive rats. *Eur J Pharmacol* **620**, 90-6 (2009)..
3. Kappert K., et al. Chronic treatment with losartan results in sufficient serum levels of the metabolite EXP3179 for PPARgamma activation. *Hypertension* **54**, 738-743 (2009).
4. Yan Y.D., et al. The physicochemical properties, in vitro metabolism and pharmacokinetics of a novel ester prodrug of EXP3174. *Mol Pharm* **7**, 2132-2140 (2010)
5. Dolber, P.C. & Spach, M.S. Picrosirius red staining of cardiac muscle following phosphomolybdic acid treatment. *Stain Techno* **62**, 23–26 (1987).
6. López, B., Querejeta, R., González, A., Beaumont, J., Larman, M. & Díez, J. Impact of treatment on myocardial lysyl oxidase expression and collagen cross-linking in patients with heart failure. *Hypertension* **53**, 236-242 (2009).
7. Livak, K.J. & Schmittgen, T.D. Analysis of relative gene expression data using real-time quantitative PCR and the 2(-Delta Delta C(T)) *Method. Methods* **25**, 402–4083 (2001).
8. López, B. *et al.* Osteopontin-mediated myocardial fibrosis in heart failure: a role for lysyl oxidase? *Cardiovasc Res* **99**, 111-120 (2013).

Supplemental Figure legends

Figure S1. Histograms represent the fold change in procollagen type I (panel A) and lysyl oxidase (LOX) (panels B) mRNA in HDFa fibroblasts stimulated without and with 10^{-6}, 10^{-5}, 10^{-4}, 10^{-3}, and 10^{-2} μg/ml TGF-β_1 for 24 hours as compared with control cells. Bars represent mean + SEM (n=5 to 8 experiments). *P<0.05 vs Control, **P<0.01 vs Control.

Figure S2. The histogram represents the fold change in the expression LOX mRNA in HDFa fibroblasts stimulated without and with 10^{-4} μg/ml TGF-β_1 for 24 hours, alone or in combination with 20 μM EXP3179, in the absence or the presence of the inhibitors of PPAR-γ (G3335, at 32 and 100 μM), and phosphatidylinositol 3-kinase (PI3K) (LY294002, at 1.4 and 10 μM), and the activators of the protein kinase-C (PKC), (Phorbol 12-myristate 13-acetate [PMA], at 0.4 and 10 μM) and cyclooxygenase 2 (lipopolysaccharide [LPS], at 1 and 50 μg/ml), as compared with control cells. Bars represent mean + SEM (n=5 to 8 experiments). * P<0.05 vs Control, † P<0.05 vs TGF-β_1, †† P<0.001 vs TGF-β_1.

Figure S3. Histograms represent the fold change in expression of LOX mRNA in HDFa fibroblasts transfected with scramble, CTFG siRNA or THBS1 siRNA incubated in the absence or the presence of 10^{-3} μg/mL TGF-β_1 (panel A) and the fold change in the expression of CTGF mRNA in HDFa fibroblasts stimulated without and with 10^{-4} μg/mL TGF-β_1 for 24 hours, in the absence or the presence of EXP3179 at 1, 2, 5, 10, 20 and 50 μM, as compared with control cells (panel B). Bars represent mean + SEM (n=5 to 8 experiments) and AU means arbitrary units. * P<0.05 vs Control, † P<0.05 vs TGF-β_1.

Table S1. Differential gene expression determined by Human Fibrosis PCR-Array analysis in human fibroblasts stimulated with TGF-β_1 in the absence or presence of EXP3179

**Symbol**	**Description**	**TGF-β1**<br>Expression vs control<br>(fold change)	**TGF-β1 + EXP3179**<br>Inhibition vs TGF-β1<br>(fold change)
ACTA2	Actin α2 smooth muscle, aorta	1.36±0.26	1.01±0.04
AGT	Angiotensinogen	1.25±0.07	1.25±0.33
AKT1	AKT/PKB signalling pathway	1.11±0.04	1.07±0.14
BCL2	B-cell CLL/lymphoma 2	1.12±0.09	1.02±0.17
BMP7	Bone morphogenetic protein 7	0.75±0.40	1.66±0.90
CAV1	Caveolin 1	0.80±0.07	0.97±0.09
CCL11	Chemokine ligand 11	1.20±0.39	4.66±6.19
CCL2	Chemokine ligand 2	1.34±0.30	0.95±0.18
CCL3	Chemokine ligand 3	0.99±0.44	3.63±3.08
CCR2	Chemokine receptor 2	1.26±0.89	3.63±2.62
CEBPB	CCAAT/enhancer binding protein-β	1.05±0.29	0.93±0.47
COL1A2	Collagen type I, alpha 2	1.11±0.19	0.82±0.20
COL3A1	Collagen type III, alpha 1	0.84±0.14	0.73±0.34
**CTGF**	**Connective tissue growth factor**	**3.20±0.79**	**1.91±0.18**
CXCR4	Chemokine receptor 4	36.7±32.7	1.35±2.25
DCN	Decorin	0.89±0.03	1.05±0.29
EDN1	Endothelin-1	2.02±0.16	1.44±0.40
EGF	Epidermal growth factor	3.95±4.87	0.88±0.40
ENG	Endoglin	0.97±0.10	1.02±0.36
GREM1	Gremlin-1	1.93±0.44	1.53±0.42
HGF	Hepatocyte growth factor	1.04±0.07	1.07±0.34
IFNG	Interferon, gamma	1.17±0.08	0.66±0.22
IL10	Interleukin 10	2.11±2.18	1.07±1.49
IL13	Interleukin 13	0.79±0.54	0.46±0.31
IL13RA2	Interleukin 13 receptor, alpha2	1.14±0.14	1.30±0.23
IL1A	Interleukin 1, alpha	0.56±0.47	0.33±0.16
IL1B	Interleukin 1, beta	1.27±0.64	1.56±1.35
IL4	Interleukin 4	0.32±0.33	1.87±2.58
IL5	Interleukin 5	1.44±1.28	0.63±0.42
ILK	Integrin-linked kinase	1.13±0.18	0.83±0.08
INHBE	Inhibin, beta E	1.82±2.12	0.04±0.01
ITGA1	Integrin, alpha 1	2.36±0.20	1.09±0.14
ITGA2	Integrin, alpha 2	0.98±0.14	1.06±0.30
ITGA3	Integrin, alpha 3	1.45±0.17	1.33±0.22
ITGAV	Integrin, alpha V	1.04±0.34	1.18±0.52
ITGB1	Integrin, beta 1	1.25±0.06	1.67±0.29
ITGB3	Integrin, beta 3	0.73±0.04	1.27±0.27
ITGB5	Integrin, beta 5	1.63±0.08	0.92±0.13
ITGB6	Integrin, beta 6	1.07±0.81	3.57±3.20
ITGB8	Integrin, beta 8	1.26±0.15	1.28±0.49
JUN	Jun proto-oncogen	1.46±0.19	1.03±0.42
**LOX**	**Lysyl oxidase**	**1.63±0.11**	**1.57±0.55**
LTBP1	Latent transforming growth factor	1.71±0.14	1.16±0.20
MMP1	Matrix metallopeptidase 1	1.04±0.08	1.04±0.13
MMP13	Matrix metallopeptidase 13	1.48±1.23	2.75±2.79
MMP14	Matrix metallopeptidase 14	1.46±0.36	1.56±0.59
MMP2	Matrix metallopeptidase 2	1.09±0.11	1.23±0.22

MMP3	Matrix metallopeptidase 3	0.83±0.07	0.97±0.13
MMP8	Matrix metallopeptidase 8	1.55±2.27	0.97±0.83
MMP9	Matrix metallopeptidase 9	0.98±1.00	1.15±0.71
MYC	V-myc myelocytomatosis viral oncogene homolog	1.25±0.08	1.07±0.03
NFKB1	Nuclear factor of kappa light polypeptide gene enhancer in B-cells	1.04±0.23	0.98±0.23
PDGFA	Platelet-derived growth factor alpha polypeptide	1.07±0.16	0.85±0.12
PDGFB	Platelet-derived growth factor beta polypeptide	2.74±1.91	1.29±0.42
PLAT	Plasminogen activator, tissue	0.78±0.34	0.80±0.24
PLAU	Plasminogen activator, urokinase	1.31±0.09	1.29±0.35
PLG	Plasminogen	2.04±2.42	0.96±0.65
SERPINA1	Serpin peptidase inhibitor 1, clade A	1.04±1.43	0.66±0.01
SERPINE1	Serpin peptidase inhibitor 1, clade E	2.45±0.04	1.43±0.11
SERPINH1	Serpin peptidase inhibitor 1, clade H	1.40±0.14	1.00±0.09
SMAD2	SMAD family member 2	0.87±0.01	1.09±0.13
SMAD3	SMAD family member 3	0.78±0.01	0.94±0.04
SMAD4	SMAD family member 4	0.93±0.03	1.18±0.20
SMAD6	SMAD family member 6	0.96±0.05	0.91±0.42
SMAD7	SMAD family member 7	1.16±0.17	1.34±0.49
SNAI1	Snail homolog 1	0.91±0.14	0.59±0.24
SP1	Sp1 transcription factor	1.07±0.08	0.96±0.07
STAT1	Signal transducer and activator of transcription 1	1.24±0.17	1.07±0.27
STAT6	Signal transducer and activator of transcription 1	1.02±0.20	1.05±0.20
TGFB1	Transforming growth factor 1	1.12±0.09	1.17±0.18
TGFB2	Transforming growth factor 2	0.48±0.12	0.81±0.05
TGFB3	Transforming growth factor 3	1.31±0.15	1.08±0.16
TGFBR1	Transforming growth factor receptor 1	1.33±0.20	1.25±0.19
TGFBR2	Transforming growth factor receptor 2	1.28±0.18	1.05±0.04
TGIF1	TGFB-induced factor homeobox 1	1.21±0.20	1.09±0.11
**THBS1**	**Thrombospondin 1**	**2.65±0.33**	**1.81±0.27**
THBS2	Thrombospondin 2	0.87±0.06	1.08±0.30
TIMP1	TIMP metallopeptidase inhibitor 1	0.98±0.13	0.83±0.19
TIMP2	TIMP metallopeptidase inhibitor 2	1.06±0.10	1.03±0.15
TIMP3	TIMP metallopeptidase inhibitor 3	0.94±0.27	2.34±0.26
TIMP4	TIMP metallopeptidase inhibitor 4	0.45±0.21	0.70±0.21
TNF	Tumor necrosis factor	1.15±0.49	1.12±1.50
VEGFA	Vascular endothelial growth factor A	1.20±0.18	0.69±0.08

Data are expressed as mean ± SD

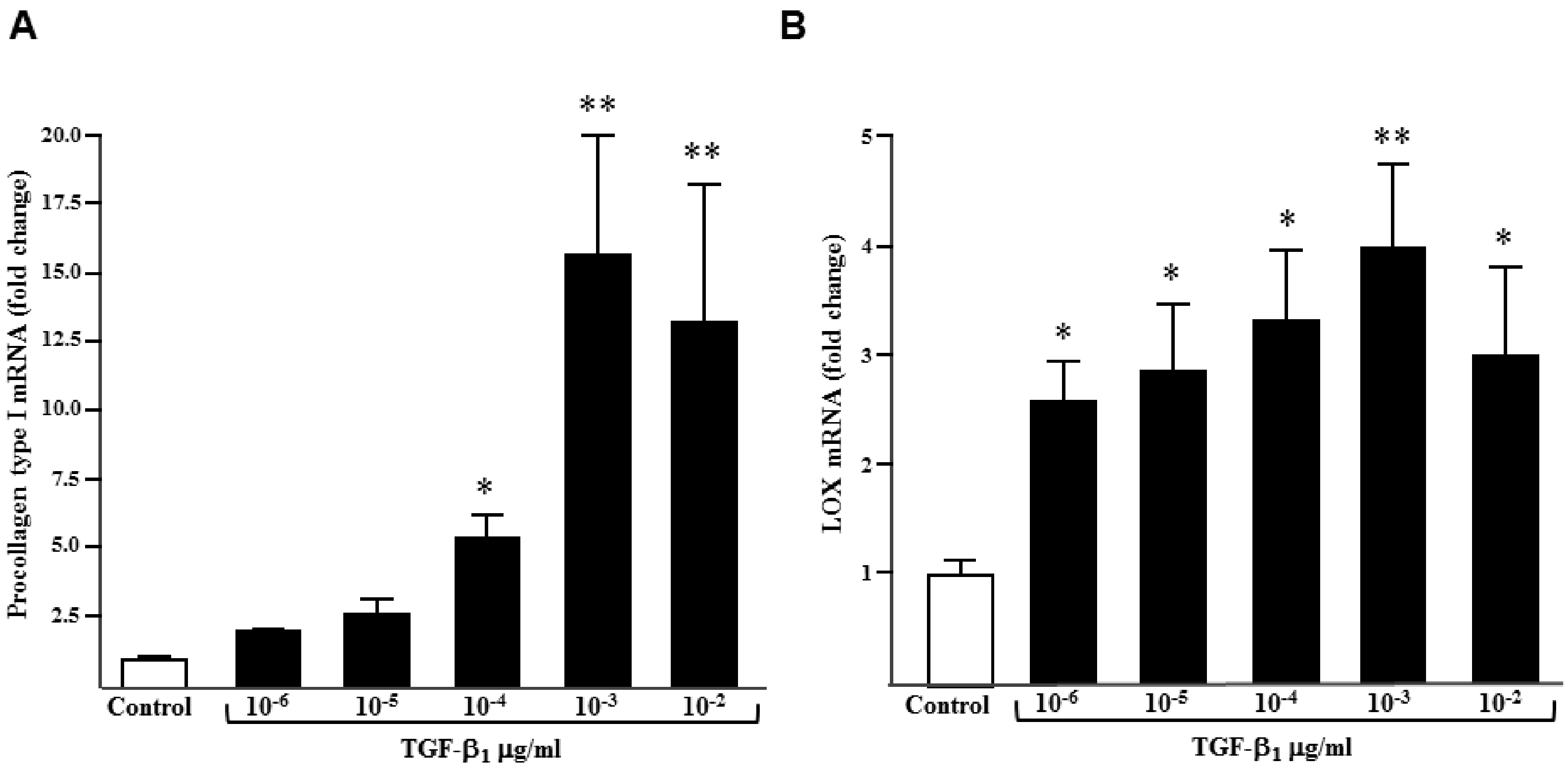
A
B
Procollagen type I mRNA (fold change)
20.0
17.5
15.0
12.5
10.0
7.5
5.0
2.5
Control
10-6
10-5
10-4
10-3
10-2
TGF-β1 μg/ml
LOX mRNA (fold change)
5
4
3
2
1
Control
10-6
10-5
10-4
10-3
10-2
TGF-β1 μg/ml

Fig. S1

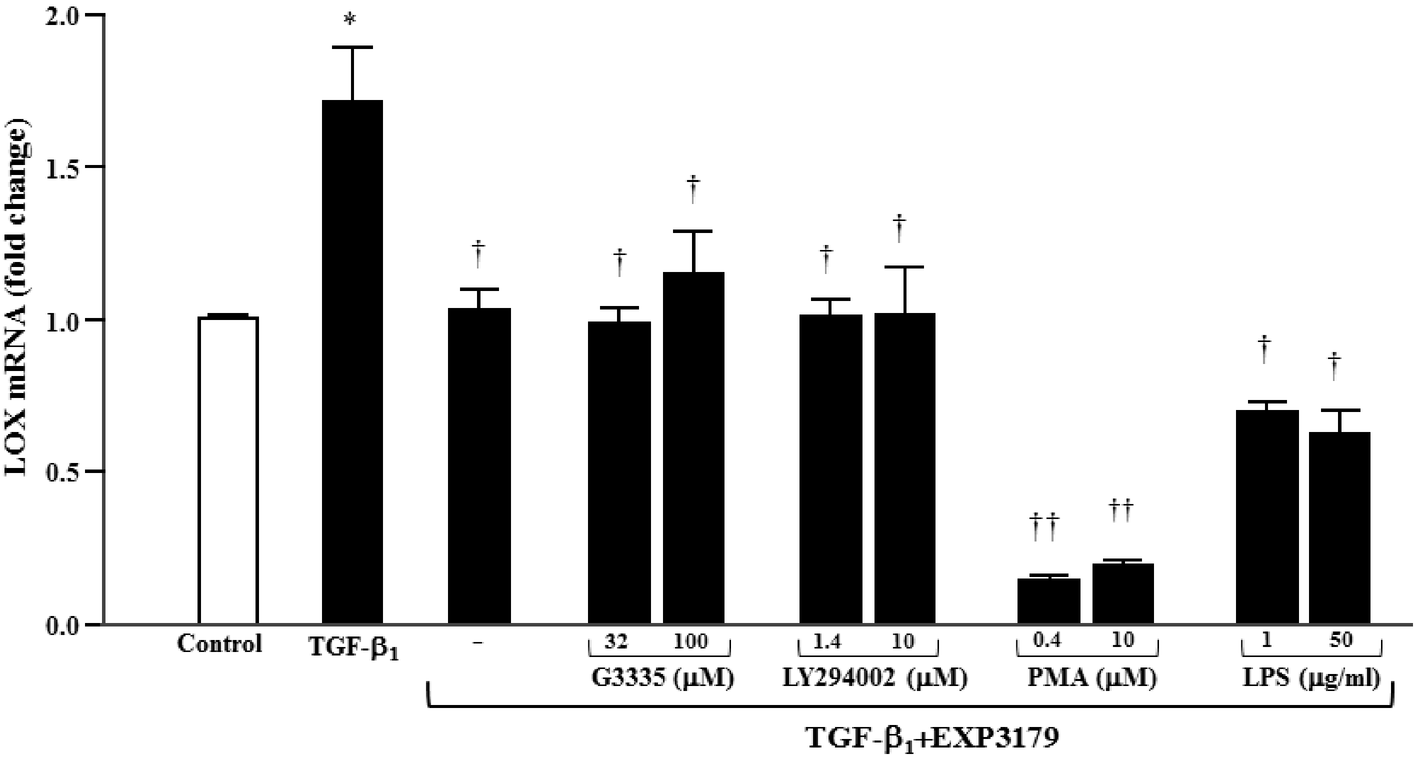
LOX mRNA (fold change)
2.0
1.5
1.0
0.5
0.0
*
†
††
Control
TGF-β1
-
32
100
G3335 (μM)
1.4
10
LY294002 (μM)
0.4
10
PMA (μM)
1
50
LPS (μg/ml)
TGF-β1+EXP3179

Fig. S2

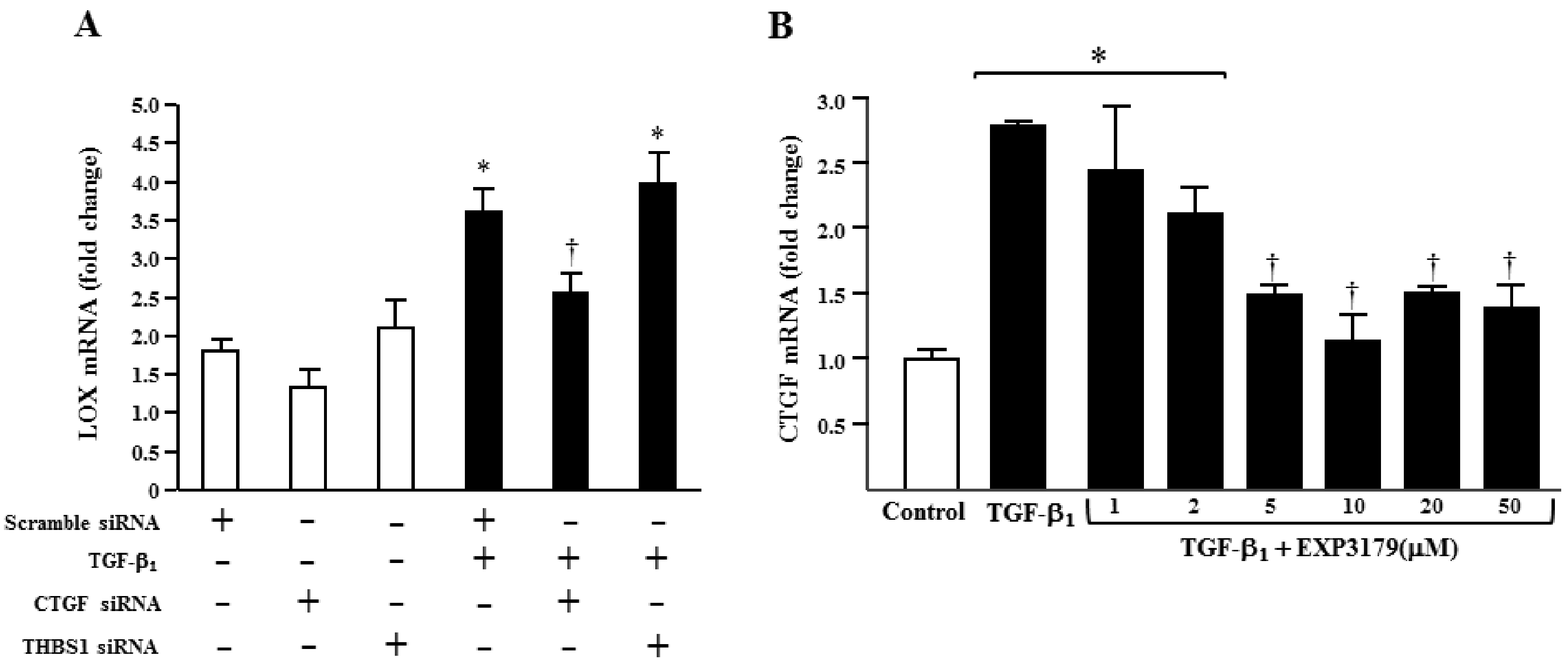
A
LOX mRNA (fold change)
5.0
4.5
4.0
3.5
3.0
2.5
2.0
1.5
1.0
0.5
0
*
†
*
Scramble siRNA + − − + − −
TGF-β1 − − − + + +
CTGF siRNA − + − − + −
THBS1 siRNA − − + − − +
B
*
CTGF mRNA (fold change)
3.0
2.5
2.0
1.5
1.0
0.5
† † † †
Control
TGF-β1
1
2
5
10
20
50
TGF-β1 + EXP3179(μM)

Fig. S3
